# Calibration of Cone Factor in Cone Penetration Test for Evaluating the Undrained Shear Strength of Silty Clay

**DOI:** 10.3390/ma18061283

**Published:** 2025-03-14

**Authors:** Caihong Wu, Yue Song, Jialin Dai, Lin Li, Xiaoqiang Gu

**Affiliations:** 1 China Three Gorges Corporation, Wuhan 430010, China; 2 Department of Geotechnical Engineering, Tongji University, Shanghai 200070, China; 3 Science and Technology Research Institute, China Three Gorges Corporation, Beijing 100053, China; 4 School of Highway, Chang’an University, Xi’an 710064, China

**Keywords:** cone penetration test, vane shear test, cone factor, cavity expansion method

## Abstract

Cone penetration test often uses the cone factor to calculate the undrained shear strength of silty clay base on the cone tip resistance data, but the accurate determination of the cone factor is challenging due to its wide range of values. This study conducted a laboratory cone penetration test and vane shear test to investigate and calibrate the cone factor for evaluating the undrained shear strength of silty clay at various depths. The cone factor is first identified based on the laboratory cone penetration test and vane shear test, and it is compared with the cone factor generated from the cavity expansion theory. Cone factor calibration has been performed by integrating laboratory model tests with the cavity expansion method, unlike conventional approaches relying on singular methodologies. The proposed cone factor is validated by the in situ undrained shear strength of Shanghai silty clay based on the in situ cone penetration test data. The results indicate that the cone factor varies significantly, ranging from 3 to 27. The cone factor from laboratory results shows good agreement with that based on the cavity expansion method. The calibrated cone factor predicts reasonable undrained shear strength measured from in situ tests. The refining method enables ±30% accuracy in predicting field-measured undrained shear strength values, establishing region-specific guidelines for East China Sea investigations.

## 1. Introduction

The transition to cleaner energy and sustainable development has become a focal point of global attention. Clean energy is rapidly replacing traditional fossil fuels, marking an inevitable direction for future energy development. Among various clean renewable energy sources, offshore wind power has experienced rapid global growth in recent years [1,2]. Compared to onshore wind power, offshore wind power provides advantages such as more stable wind speeds, higher energy generation efficiency, and reduced noise impact. To meet the growing demand for clean energy, many countries are launching large-scale offshore wind power projects in coastal regions [3,4]. Offshore wind farm construction involves complex environmental conditions, and turbine foundation stability is critical for the safe operation of offshore wind projects [5,6]. Because turbine foundations are typically situated on cohesive seabed soils, careful consideration should be given to the bearing capacity and shear strength of the foundation soil during design and construction. This is essential to ensure the stability of turbine foundations and the long-term safety of offshore wind operations.

Clay soils generally exhibit a high void ratio, high water content, and low permeability. During rapid construction on cohesive soil layers, foundation soils often remain in an undrained state [7,8]. The undrained shear strength *S_u_*, an important parameter in geotechnical engineering design, influences the maximum bearing capacity of port structures, shallow foundations, and pile foundations. The accurate measurement of *S_u_* in clay soils is critical for engineering design and construction. Common methods for determining *S_u_* include cone penetration testing (CPT), vane shear testing (VST), and triaxial tests. Among these, CPT is widely used for its simplicity and efficient data recording capabilities [9,10,11,12]. *S_u_* values along the depth profile can be calculated using cone tip resistance *q_c_* and the cone factor *N_k_* for one test. *N_k_* values vary significantly across sites. Several studies [13,14,15,16,17,18,19] have documented diverse *N_k_* values, as summarized in Table 1. The significant variability in *N_k_* values reported in prior studies (Table 1, *N_k_* = 5−57) can be attributed to site-specific soil characteristics and geological conditions. For example, high-plasticity silty clays in Sudan [13] exhibit *N_k_* = 32−39 due to strong interparticle cementation, whereas low-plasticity soft clays in Indonesia [16] show lower *N_k_* values (5–12) as structural collapse dominates under shear. Similarly, the influence of geological history is evident in Hungarian Holocene clays [18] (*N_k_* = 12−32), where stress history sensitivity contrasts sharply with over-consolidated Niger Delta clays [19] (*N_k_* = 34−57). These variations highlight the limitations of applying universal *N_k_* correlations and underscore the necessity of context-specific calibration, particularly for offshore wind foundations in heterogeneous seabeds. The cone type, shape, and soil mechanical properties can also influence *N_k_* [20,21], emphasizing the importance of studying soil characteristics and refining methods for selecting *N_k_* values.

Several methods exist for estimating the *S_u_* of clay based on CPT, including empirical relationships, numerical simulations, and model tests. Previous studies [22,23,24] established the relationship between *q_c_* and *N_k_*, as shown in Table 2. However, the empirical relationship is only applicable to specific soil conditions, and it is difficult to fully reflect the complex stress state of clay in engineering. Existing methods for estimating *N_k_*, including empirical formulas and numerical simulations [25,26,27], face inherent limitations when applied to complex field conditions. Empirical approaches, such as Terzaghi’s equation [22], often oversimplify stress effects, leading to significant errors in partially drained high-OCR clays. Numerical models, while theoretically robust, frequently assume idealized plasticity and neglect strain-softening behaviors observed in sensitive marine clays [10]. These shortcomings are exacerbated in natural deposits where fabric anisotropy and fissures alter soil response. To address these gaps, our study integrates controlled laboratory modeling with field validation, explicitly accounting for strain localization and drainage effects. The range of *N_k_* can be explored through numerical simulation [25,26,27], but this method depends on selecting an appropriate constitutive model that reflects the actual stress state. In situ and laboratory modeling tests more accurately reflect the stress state and stress history of clays. During testing, the *S_u_* can be calculated from *q_c_* and *N_k_*. However, the correlation between *N_k_* results from in situ and laboratory modeling tests remains unclear and requires further investigation.

This study aims to determine the cone factor *N_k_* for CPT in silty clay through laboratory and field tests. Remolded clay samples were prepared in a model box, and CPT and VST tests were conducted under overburden stresses of 25 kPa and 50 kPa. Undisturbed soil parameters were measured through laboratory tests, and the *N_k_* coefficient was calculated accordingly. The contact force between the soil and the probe in all directions is considered when using the cavity expansion method based on the Modified Cam-Clay constitutive model. The experimental results were compared with analytical solutions derived from the cavity expansion method to validate the proposed laboratory test procedure. Additionally, field test results were analyzed to predict the *N_k_* value, further supporting the validity of the laboratory findings.

## 2. Laboratory Tests

### 2.1. Test Soil

Soil samples were collected from an offshore wind farm in the East China Sea, approximately 19.5 km from the coastline. The water depth at the site ranges from 10.2 to 12.0 m. Stratified samples were collected using thin-walled samplers such as Shelby tubes at various depths. All samples were immediately sealed with wax or tape after sampling and promptly transported to the laboratory [28,29,30]. The United States Department of Agriculture (USDA) soil textural triangle was used to classify the soil type [31,32]. The subsurface soil within the embedded depth mainly consists of clay and sandy soils. Specifically, the stratigraphy includes silty clay from 0 to 7 m, silty sand from 7 to 20 m, and silty clay from 20 to 40 m below the ground surface. The physical properties of each soil layer are listed in Table 3. This study focuses on the silty clay in third layer, located 5 m below the surface, where CPT and VST data are available.

### 2.2. Test Equipment

**Figure 1:**
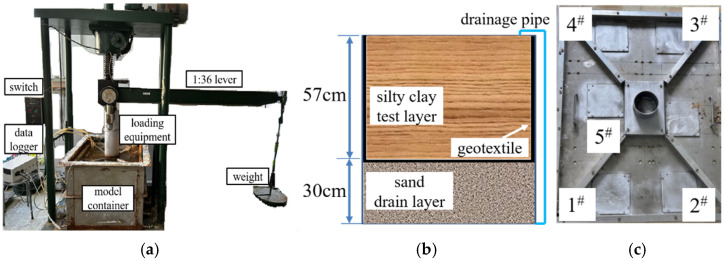
Experimental equipment: (**a**) consolidation equipment, (**b**) container size, and (**c**) loading plate.

The Tongji DGJ-150(500) large-scale oedometer, shown in Figure 1a, was used to consolidate the silty clay slurry. The equipment has four main components, which include a large-scale lever loading system, an electric lever leveling device, a control system, and a model container. The model consists of a 57 cm thick silty clay layer and a 30 cm thick sand layer, as shown in Figure 1b. A loading system, driven by a servo motor, applies a designed pressure to the silty clay in the model container through a loading plate for consolidation, as shown in Figure 1c. The loading plate includes five testing ports, numbered 1^#^ to 5^#^, for CPT and VST testing.

**Figure 2:**
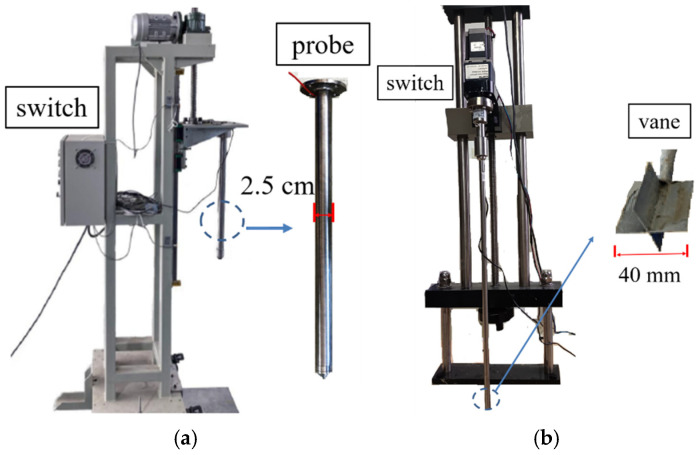
Soil testing equipment: (**a**) cone penetration test, (**b**) vane shear test.

The CPT, which was designed by University of Western Australia (UWA) in Australia, includes a probe rod, a cone tip, and a controller, as shown in Figure 2a. The probe rod has a diameter of 2.5 cm and a cross-sectional area of 4.9 cm^2^. The cone penetration tests were conducted in accordance with ASTM [33], with the cone probe penetrated at a controlled rate of (1.2 ± 0.3) m/min. As shown in Figure 2b, the VST has a vane head with a diameter of 40 mm. The laboratory vane shear tests were performed per ASTM [34] at incremental depths of 10 cm, 20 cm, and 30 cm below the loading plate. The vane rotation rate was maintained at 0.1°/s throughout testing to ensure accurate shear strength measurement. Data were acquired at 0.1° intervals, and the peak shear strength of the soil was determined.

### 2.3. Test Protocol and Procedure

The prepared clay sample, initially in a slurry state, was allowed to settle for 7 days before consolidation testing. The corresponding loading pressures are listed in Table 4. The test employed a seven-stage incremental loading method. In the first stage, soil consolidation relied mainly on the self-weight of the hanging plate and lever, while subsequent stages used additional weights to apply pressure.

## 3. Test Results

### 3.1. Soil Mechanical Behavior Monitoring

The clay samples from the Shanghai wind farm were tested using Atterberg limit tests to determine their liquid limit (LL), plastic limit (PL), and plasticity index (PI). The results showed an LL of 44%, a PL of 22.6%, and a PI of 22%. This method has been widely used in previous studies [35,36,37] to accurately determine the LL and PL of soil samples. During the preparation of remolded slurry, the in situ soil samples were air-dried, crushed, and sieved following relevant testing standards [38]. Water was then added to achieve 1.5 times the liquid limit water content for full saturation. The slurry was mixed in a vacuum mixer until fully saturated, resulting in the remolded clay slurry.

**Figure 3:**
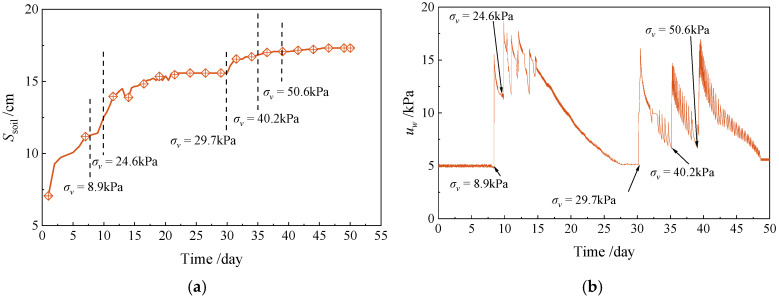
Laboratory test process monitoring: (**a**) soil settlement, (**b**) pore water pressure.

Figure 3a shows the monitoring results of soil settlement during the test. After each load application, the displacement of the loading plate was measured with an infrared rangefinder. During the early stages of the test, the settlement rate increased rapidly due to the soil’s low initial strength. As the number of loading stages increased, the strength of the clay improved, resulting in a gradual reduction in the settlement rate. Figure 3b illustrates the pore water pressure changes observed during the test. Pore water pressure increased rapidly during each incremental loading. Consolidation was deemed complete when the soil settlement remained unchanged for seven consecutive days and the pore water pressure ceased to decrease. This indicated that the soil consolidation stress had equilibrated with the applied overburden stress. CPT and VST tests were conducted at overburden stresses of 25 kPa and 50 kPa, respectively.

### 3.2. Cone Penetration Test Results

**Figure 4:**
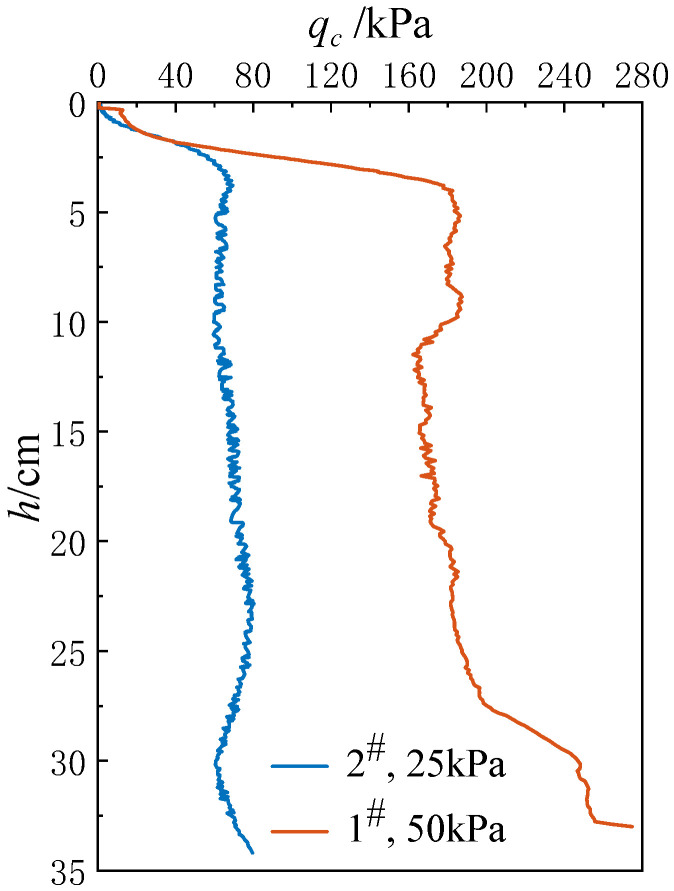
CPT results in laboratory test.

Figure 4 presents the results of the CPT laboratory tests. At an overburden stress of 25 kPa, the cone tip resistance *q_c_* at 2^#^ measured 59.5 kPa at a penetration depth *h* of 10 cm, 73.1 kPa at 20 cm, and 60.6 kPa at 30 cm. The *q_c_* values increased with increasing overburden stress. At an overburden stress of 50 kPa, the *q_c_* value at 1^#^ reached 180.6 kPa at *h* = 10 cm, 178.8 kPa at 20 cm, and 247.1 kPa at 30 cm.

### 3.3. Vane Shear Test Results

**Figure 5:**
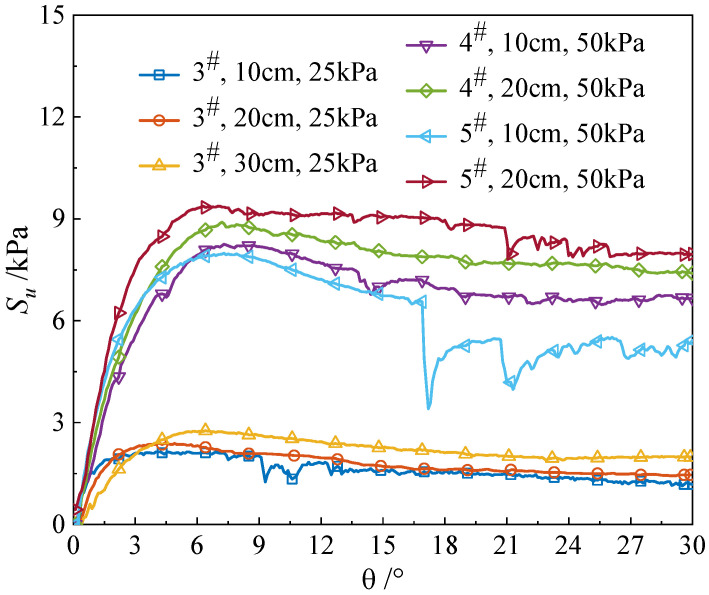
VST results in laboratory test.

Figure 5 illustrates the results of the VST tests.

In the undamaged state, the soil shows rising shear stress with strain until structural breakdown occurs, followed by strength degradation and convergence to a residual value, consistent with prior results [39,40,41]. Under an overburden stress of 25 kPa at 3^#^, the vane rotated 5.8°, 4°, and 4° at depths of 10 cm, 20 cm, and 30 cm, respectively. The corresponding peak shear strengths *S_u_* were 2.1 kPa, 2.4 kPa, and 2.7 kPa. Under an overburden stress of 50 kPa at 4^#^, the vane rotated 8.2° at a depth of 10 cm, and the measured *S_u_* was 7.9 kPa. Under a depth of 20 cm, the vane rotated 7.2°, and the measured *S_u_* was 8.4 kPa. At 5^#^, the vane rotated 7.4° at a depth of 10 cm, and the measured *S_u_* was 7.9 kPa. At a depth of 20 cm, the vane rotated 6.6°, and the measured *S_u_* was 9.4 kPa. The VST results indicate that soil shear strength increases with rising overburden stress and with greater test depths at the same location.

### 3.4. Cone Tip Coefficient Analysis

Tests were conducted under different effective overburden stresses to obtain CPT and VST data at various depths. The purpose was to analyze the variation of the cone factor *N_k_*. The *N_k_* can be defined as $(q_{c}-\sigma_{\nu 0})/S_{u}$, where $\sigma_{v0}$ is the total overburden stress of soil [23]. The detailed results are summarized in Table 5. Under an overburden stress of 25 kPa, the results from 2^#^ and 3^#^ indicate an average *N_k_* of 13.5 for the peak strength. Under an overburden stress of 50 kPa, the results from 1^#^ and 4^#^ show a fitted *N_k_* of 15.1.

### 3.5. Comparison of Laboratory N_k_ with Cavity Expansion Method

**Figure 6:**
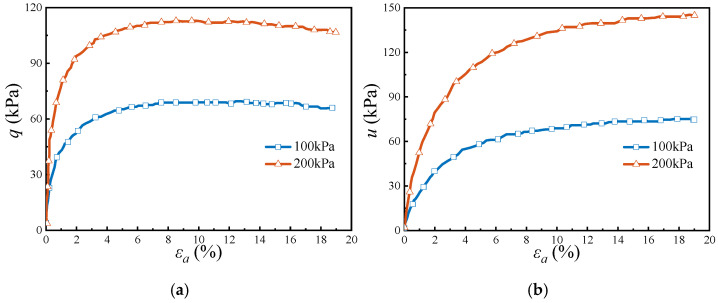
Undrained triaxial test results from laboratory test results: (**a**) deviatoric stress—axial strain relation and (**b**) pore water pressure—axial strain relation.

Undrained triaxial shear tests were performed on undisturbed soil samples. The deviatoric stress-axial strain and pore water pressure-axial strain relationships obtained from drained triaxial tests are illustrated in Figure 6. The results indicate that all specimens show a softening response. As axial strain increases, the deviatoric stress rises until it reaches a peak, occurring at an axial strain of around 6%. The peak deviatoric stress values are 69.25 kPa and 112.46 kPa under confining pressures of 100 kPa and 200 kPa, respectively. In contrast to the deviatoric stress, pore water pressure exhibits a continuous increase with axial strain, as depicted in Figure 6b.

Based on the spherical cavity expansion analysis in a modified Cam-Clay [42,43], the pore pressure *u* and ultimate cavity expansion stress $\sigma_{u}$ at the cavity wall are expressed as

(1) $$u=q_{t}-\frac{2}{3}Mp_{0}^{\prime }{\left(\frac{R}{2}\right)}^{\Lambda }-p_{0}^{\prime }{\left(\frac{R}{2}\right)}^{\Lambda }$$

(2) $$\sigma_{u}=p_{0}+\frac{m}{\sqrt{3^{m}}}Mp_{0}^{\prime }{\left(\frac{R}{2}\right)}^{\Lambda }\left\{1+\ln\left[\frac{G\sqrt{m+2}}{Mp_{0}^{\prime }{\left(R/2\right)}^{\Lambda }}\right]\right\}$$

(3) $$M=\frac{6\sin\phi^{\prime }}{3-\sin\phi^{\prime }}$$

where *R*, *m* are constants. Note that equations are the exact closed-form solutions for *R* = 2 or an elastic-perfectly plastic material in the context of the MCC model. *m* = 1 for the cylindrical case and *m* = 2 for the spherical case. *G* is the shear modulus. For a spherical cavity in an MCC material, G can be expressed as a function of the specific volume υ. *p*_0_ is the initial mean stress. *M* is the slope of the critical state line, defined as $6\sin\phi^{\prime }/(3-\sin\phi^{\prime })$, and $\phi^{\prime }$ is the effective friction angle. Λ is the plastic volumetric strain ratio, defined as (1−κ/λ). λ is the slope of the unloading–reloading line in the $\upsilon –\ln p^{\prime }$ plane. κ is the slope of the normal compression line on the $\upsilon –\ln p^{\prime }$ plane. Although the value of Λ may be as high as 1.0 for some cases [44], a typical value of Λ of 0.75 is assumed in subsequent analyses. Elastic-perfectly plastic behavior when *R* = 2. $p_{0}^{\prime }$ is the effective mean stress. Chang et al. [43] considered only the influence of ultimate cavity expansion stress on cone tip resistance, neglecting the forces acting on the cone tip from various directions. Ladanyi et al. [45] mentioned that at failure, the shear strength of the soil across the entire cone or wedge area is fully mobilized, and the following relationship was derived based on static equilibrium. The cone tip resistance equation, which accounts for shear strength from various directions, is expressed as follows:

(4) $$q_{c}=\sigma_{u}(1+\tan\phi^{\prime })+c$$

where *c* is the cohesion of silty clay. Table 6 lists the parameters obtained from the laboratory triaxial tests under different confining stresses. Note that *S_u_* is the peak the undrained shear strength. The *E*, *c*, and $\phi^{\prime }$ were calculated according to Yin et al. [46].

**Figure 7:**
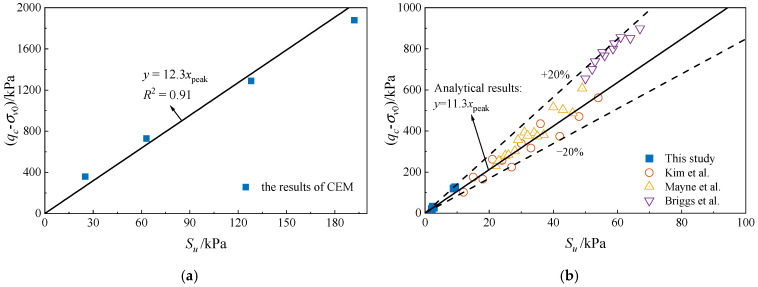
Comparison of *N_k_* results: (**a**) fitting line for the data from CEM, (**b**) comparison between *N_k_* from laboratory tests [20,47,48] and analytical solution.

Figure 7a illustrates the analytical results of the cavity expansion method (CEM), with a fitted *N_k_* value of 12.3. Figure 7b compares the *N_k_* values from the CEM with those obtained from laboratory model tests on silty clay in the third layer. As seen in Figure 7b, *N_k_* values are compared with other studies [20,47,48] for silty clay. All the *N_k_* data points differ by less than 20% from the CEM results. The results indicate that the *N_k_* values from the laboratory tests approximately match those from the CEM. This demonstrates the feasibility of using laboratory model tests to predict *N_k_*.

## 4. Field Measurement Results

### 4.1. The Results of In Situ Tests

CPT and VST in situ tests were carried out in the wind farm. As shown in Table 7, the distance between the CPT and VST test points is within 2 km. All survey points are characterized as silty clay within the depth range of 0 to 7 m, and the saturated bulk density difference between the test sites is less than 10%.

**Figure 8:**
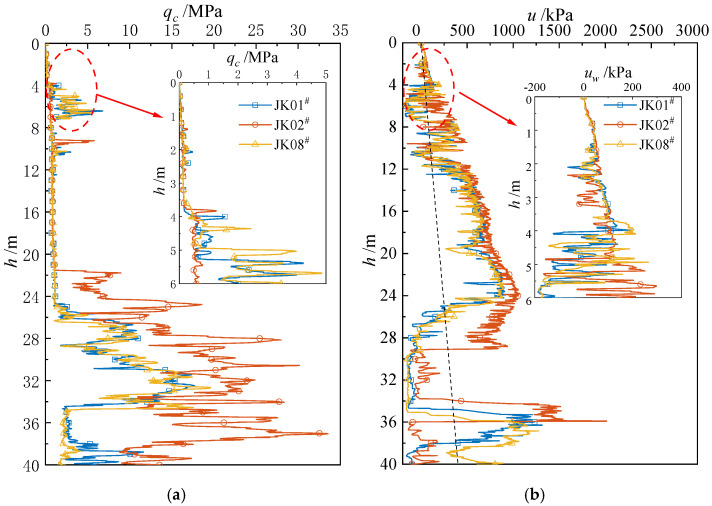
In situ CPTU test results: (**a**) cone tip resistance, (**b**) pore water pressure.

Figure 8 presents the CPTu in situ test results for locations JK01 ^#^, JK02 ^#^, and JK08 ^#^. As shown in Figure 8a, *q_c_* increases gradually up to a penetration depth of 10 m. At a depth of 2.5 m, the *q_c_* values for JK01^#^, JK02^#^, and JK08^#^ are all 0.14 MPa. At a depth of 5 m, the *q_c_* values are 0.64 MPa, 0.73 MPa, and 0.44 MPa for JK01^#^, JK02^#^, and JK08^#^, respectively. Between a depth of 10 m and 20 m, *q_c_* remains nearly constant. When the depth is beyond 20 m, *q_c_* increases significantly. Figure 8b presents the pore water pressure results, which indicate that u increases with a depth up to 25 m. At a depth of 2.5 m, the pore water pressures *u* for JK01^#^, JK02^#^, and JK08^#^ are 83 kPa, 66 kPa, and 81 kPa, respectively. At a depth of 5 m, the *u* is 89 kPa, 122 kPa, and 85 kPa.

**Figure 9:**
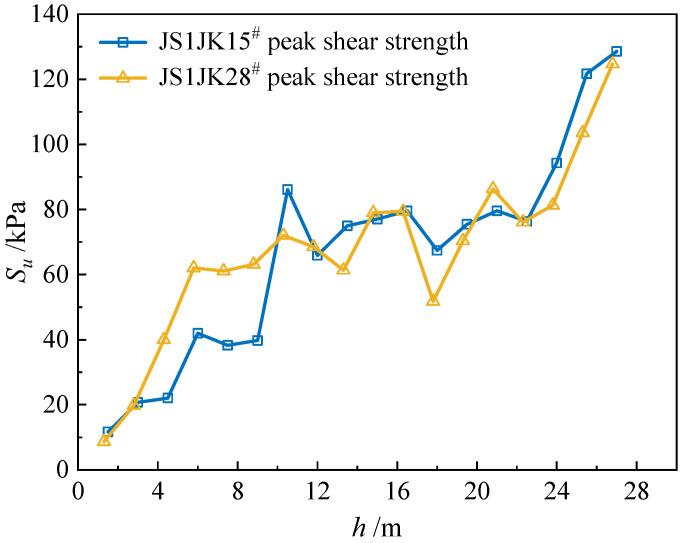
In situ VST test results.

Figure 9 illustrates the in situ VST test results. At depths up to 2 m, the peak shear strengths *S_u_* of the soil at both test locations differ by less than 10%.

At a depth of 2.5 m, the average *S_u_* is 13.3 kPa. At a depth of 5 m, the average *S_u_* is 21.9 kPa. Between depths of 12 m and 22 m, the variations in *S_u_* at the two test locations remain small, within 10%. At depths exceeding 22 m, the *S_u_* at both test locations increase significantly.

**Figure 10:**
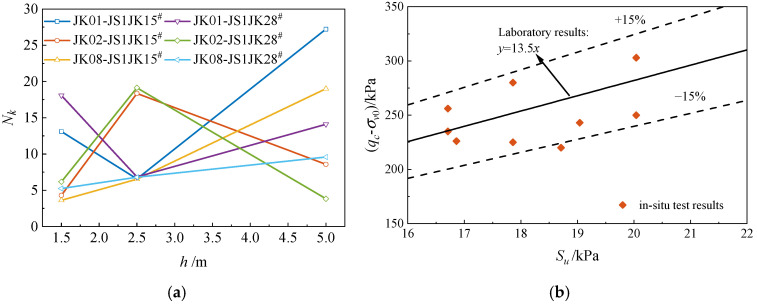
In situ test results: (**a**) *N_k_* coefficient in situ test results, (**b**) comparison of *N_k_* results.

Figure 10a illustrates the calculated values of the cone factor *N_k_*, which range from 5 to 26.

At a test depth of 1.5 m, the average *N_k_* value is 12.5. At a depth of 2.5 m, the average *N_k_* value decreases slightly to 11.8. However, at a depth of 5 m, the average *N_k_* value increases to 17.8. Figure 10b compares the *N_k_* values obtained from laboratory tests and in situ tests. Most data points deviate by less than 15% from the in situ test results. The results indicate good agreement between the model tests and in situ tests.

### 4.2. Cone Factor Evaluation

**Figure 11:**
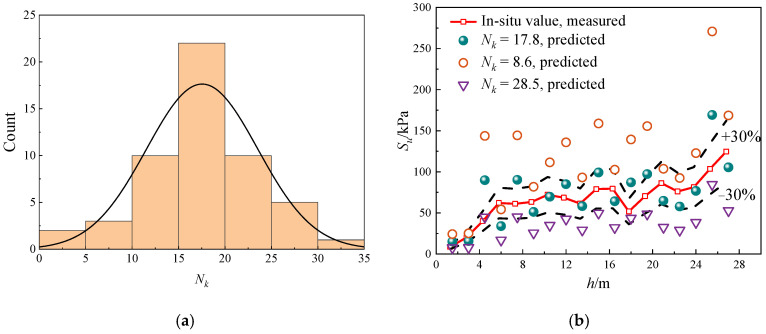
Evaluation of cone factor: (**a**) the statistical analysis of *N_k_* variability, (**b**) sensitivity analysis of cone factor to undrained shear strength.

As shown in Figure 11a, the laboratory and in situ *N_k_* values yield a mean of 17.8 and a coefficient of variation (COV) of 13.6%, which falls within the moderate-low variability range for silty clays as reported by Salgado et al. [49]. The sensitivity analysis of *N_k_* to *S_u_*, and the results, are shown in Figure 11b. The difference between the VST and CPT data at each point was less than 10%. The in situ data of JS1JK28^#^ were selected for *S_u_* measured values, and the data of JK02^#^ were selected for CPT prediction. As can be seen from the figure, the predicted value of *S_u_* decreases with the increase in *N_k_*. When *N_k_* is 17.8, the predicted value of *S_u_* is closer to the measured value. Most of the predicted values differ by less than 30% from the in-situ values.

**Figure 12:**
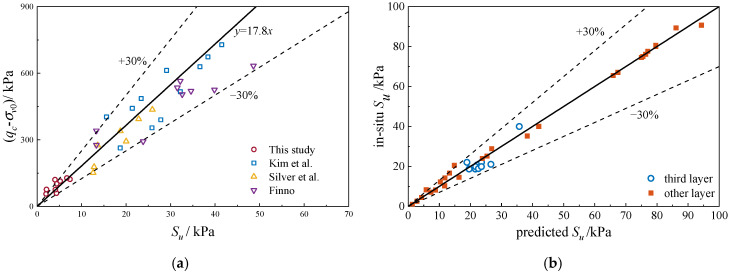
Comparison of undrained shear strength values: (**a**) comparison of this study with other studies [15,51,52], (**b**) comparison of measured and predicted undrained shear strength values.

This study estimated soil shear strength by integrating *N_k_* with CPT in situ test results. Given the inherent uncertainties arising from sample heterogeneity, Salgado [50] demonstrates that theoretical predictions of penetration resistance within ±30% of experimental values are considered acceptable in engineering practice. Figure 12a compares *N_k_* value with other studies [15,51,52] for silty clay. The results show the deviation is within ±30%. The predicted values were compared with the measured values from in situ tests at various depths, as shown in Figure 12b. The results show that the differences between the predicted and measured values are within ±30%.

## 5. Discussion

This study establishes a novel framework integrating laboratory model tests and cavity expansion theory to calibrate the cone factor *N_k_* for silty clays in the East China Sea. The key findings demonstrate that *N_k_* values derived from laboratory experiments and theoretical predictions align closely with field measurements, validating the reliability of the proposed methodology. The calibrated *N_k_* = 17.8 enables the accurate estimation of undrained shear strength *S_u_* from cone penetration test (CPT) data, addressing a gap in offshore wind foundation design for region-specific geotechnical conditions. The combination of laboratory and theoretical approaches overcomes the oversimplifications of purely empirical *N_k_* correlations [22,23,24], offering a mechanistic basis for *S_u_* prediction. The nature of clay varies from region to region, and *N_k_* values vary greatly. The observed *N_k_* range (3–27) reflects the inherent variability of silty clays, consistent with prior studies reporting *N_k_* = 7–20 for Korean marine clays [15] but contrasting sharply with *N_k_* = 34–57 in Niger clays [19]. This also reflects that this study is applicable to the clay of the East China Sea.

The study’s novelty lies in coupling physical modeling with the elastoplastic cavity expansion method to analyze multi-directional soil–cone interactions, a significant advancement over conventional single-method calibrations. However, simplifications in laboratory conditions, such as idealized stress histories and remolded samples, may underestimate natural fabric anisotropy. The modified Cam-Clay model used in this study assumes isotropic elasticity, neglecting strain-rate effects evident in field CPTs, which could explain minor *S_u_* underestimations at depths above 20 m. Additionally, sample disturbance during slurry consolidation may reduce sensitivity compared to undisturbed marine clays. Future research should consider three aspects of improvement. Firstly, laboratory tests under varied overburden stresses (e.g., 50–300 kPa) are essential to establish explicit correlations between *N_k_* and depth-dependent stress states in silty clays, particularly elucidating how *N_k_* changes under high-stress regimes. Secondly, enhancing the cavity expansion method to account for transverse anisotropy and shear moduli will better capture directional cone resistance discrepancies observed between natural and remolded clays. Thirdly, the systematic collection of in situ CPT and VST data across all soil layers is critical to establish depth-dependent *N_k_* correlations.

## 6. Conclusions

This study established a novel theoretical-experimental framework to determine the cone factor *N_k_* for silty clay in the East China Sea. The methodology integrates laboratory tests with a modified Cam-Clay-based cavity expansion theory, explicitly simulating multi-directional stress during cone penetration. Furthermore, the derived *N_k_* values were validated against field conditions, enabling a CPT calibration framework to predict shallow-layer undrained shear strength *S_u_*.

1. *N_k_* determination combines the cavity expansion method and laboratory tests. The cavity method, incorporating soil shear strength and soil–cone interaction kinematics, yields *N_k_* values aligning with laboratory tests. This consistency confirms the reliability of both approaches: the theoretical simulation of axial-radial stress complements the experimental replication of directional penetration effects, enhancing *N_k_* accuracy.
2. Laboratory tests show that cone tip resistance *q_c_* and undrained shear strength *S_u_* increase with overburden stress, with the effective stress principle governing cohesive soil. The ratio of *N_k_* values corresponding to peak strength in in situ and laboratory tests is 0.91, suggesting that *N_k_* values from laboratory tests can reliably calculate *S_u_* for silty clay.
3. For the silty clay in third layer, the *N_k_* values from laboratory model tests are comparable to those from in situ tests. The predicted shear strength at various depths based on laboratory *N_k_* values differs from in situ measurements within ±30%. This shows that laboratory tests can reliably estimate in situ soil shear strength and support engineering investigations effectively.

## Figures and Tables

**Table 1:** Typical values of cone factor *N_k_* for various soil types.

Soil Type	Source	Characteristics	*N_k_* Range	Reference
Silty clay	Sudan	High plastic	32–39	Ismail and Zein [13]
Clay	Malaysia	High plastic	12–19	Abdel Rahman [14]
Silty clay	Korea	High plastic, soft marine clay	7–20	Kim et al. [15]
Clay	Indonesia	Soft clays, high plastic	5–12	Chen [16]
Clay	Germany	Soft clays	8–29	Gebreselassie [17]
Clay	Hungary	Soft Holocene clays	12–32	Rémai [18]
Clay	Niger	Soft to firm saturated clays	34–57	Otoko et al. [19]

**Table 2:** *N_k_* analysis method.

Equation	Reference
$S_{u}=\frac{q_{c}}{N_{k}}$	Terzaghi et al. [22]
$\begin{array}{c} S_{u}=\frac{q_{c}-\sigma_{v0}}{N_{k}} \end{array}$	Yu et al. [23]
$q_{c}^{\prime }=N_{k}\sigma_{v0}^{\prime }$	Yi et al. [24]

Note: $\sigma_{v0}$ is the total overburden stress of soil, $\sigma_{v0}^{\prime }$ is the effective overburden stress of soil, and $q_{c}^{\prime }$ is the effective cone-tip resistance.

**Table 3:** Physical property index of each soil layer.

Soil Type	Depth *h* (m)	Saturated Density *γ*_sat_ (kN·m^−3^)	Effective Density *γʹ* (kN·m^−3^)
Silty clay	0–7	21.55	11.74
Sandy silt	7–20	23.32	13.51
Silty clay	20–40	22.67	12.86

**Table 4:** DGJ-150(500) lever consolidation instrument pressure comparison table.

Loading Lever	Weight (kg)	*σ_v_*_0_ (kPa)
1	5	3.69
2	10	8.91
3	10	14.12
4	20	24.55
5	10	29.76
6	20	40.19
7	20	50.62

**Table 5:** Results of CPT tests.

	2^#^ and 3^#^	2^#^ and 3^#^	2^#^ and 3^#^	1^#^ and 4^#^	1^#^ and 4^#^
*h* (cm)	10	20	30	10	20
*σ_v_*_0_ (kPa)	27.2	29.2	31.3	52.2	54.2
$\sigma_{v0}^{\prime }$ (kPa)	26.2	27.2	28.3	51.2	52.2
*S_u_* (kPa)	2.1	2.4	2.7	7.9	9.4
*q_c_* (kPa)	57.5	61.4	58.8	182.6	178.8
*N_k_*	14.9	14.2	11.3	16.6	13.5

**Table 6:** Parameters for spherical cavity expansion analysis.

*P*_0_ (kPa)	*S_u_* (kPa)	*E* (MPa)	*ν*	*c* (kPa)	$\phi^{\prime }$ (°)
50	25	4.7	0.3	8.3	30.6
100	63	6.8	0.3	8.3	30.6
200	128	9.6	0.3	8.3	30.6
300	202	11.2	0.3	8.3	30.6

**Table 7:** Survey sites information.

Number	Plane X-Coordinate (m)	Plane Y-Coordinate (m)	Soil Type	Depth (m)	Saturated Density *γ*_sat_ (kN·m^−3^)	Test
JK01^#^	3,394,812.3	356,848.8	Silty clay	0–7	21.55	CPT
JK02^#^	3,394,799.5	356,902.5	Silty clay	0–7	22.32	CPT
JK08^#^	3,392,216.5	357,409.3	Silty clay	0–7	21.86	CPT
JS1JK15^#^	3,389,312.6	357,312.6	Silty clay	0–7	21.12	VST

## Data Availability

The original contributions presented in the study are included in the article, further inquiries can be directed to the corresponding author.
